# Habitual night waking associates with dynamics of waking cortical theta power in infancy

**DOI:** 10.1002/dev.22344

**Published:** 2022-11-21

**Authors:** Louisa K. Gossé, Frank Wiesemann, Clare E. Elwell, Emily J. H. Jones

**Affiliations:** ^1^ Centre for Brain and Cognitive Development, Birkbeck University of London London UK; ^2^ Research & Development Procter & Gamble Schwalbach am Taunus Germany; ^3^ Department of Medical Physics and Biomedical Engineering, Biomedical Optics Research Laboratory University College London London UK

**Keywords:** actigraphy, EEG, infant, night waking, theta power, theta power change

## Abstract

The implications of the substantial individual differences in infant sleep for early brain development remain unclear. Here, we examined whether night sleep quality relates to daytime brain activity, operationalized through measures of EEG theta power and its dynamic modulation, which have been previously linked to later cognitive development. For this longitudinal study, 76 typically developing infants were studied (age: 4–14 months, 166 individual study visits) over the course of 6 months with one, two, three, or four lab visits. Habitual sleep was measured with a 7‐day sleep diary and actigraphy, and the Brief Infant Sleep Questionnaire. Twenty‐channel EEG was recorded while infants watched multiple rounds of videos of women singing nursery rhymes; oscillatory power in the theta band was extracted. Key metrics were average theta across stimuli and the slope of change in theta within the first novel movie. Both objective and subjective sleep assessment methods showed a relationship between more night waking and higher overall theta power and reduced dynamic modulation of theta over the course of the novel video stimuli. These results may indicate altered learning and consolidation in infants with more disrupted night sleep, which may have implications for cognitive development.

## 1 INTRODUCTION

Across the first 2 years of life, infants transition from sleeping up to 19 hours a day in short chunks with no diurnal rhythm to a consolidated 10–12 h at night, with a single nap in the day (Bathory & Tomopoulos, 2017). Overlaid on these broad developmental trends, there are large individual differences in infant sleep and especially in sleep consolidation (Bathory & Tomopoulos, 2017). These individual differences are important to understand as sleep has been shown to reflect and play an important role in brain and cognitive development (Gorgoni et al., 2020; Mason et al., 2021). For instance, longer sleep duration has been associated positively with developmental status in 10‐ to 13‐month‐olds (Gibson et al., 2012; Scher, 2005), with stronger problem‐solving abilities (Horger, DeMasi, et al., 2021), higher preference for faces (Sun et al., 2016), or better executive functioning later on (Bernier et al., 2013). While sleep duration has been extensively studied, understanding of the role of specific sleep parameters such as sleep fragmentation (i.e., time spent awake at night [WASO], number of night awakenings as signaled to the parent) is more limited. Preliminary evidence suggests that sleep fragmentation, too, can predict developmental status and later cognitive outcomes (Huhdanpää et al., 2019; Pisch et al., 2019). Further, disturbances in sleep are observed in children with neurodevelopmental conditions (Angriman et al., 2015) and emerge from infancy (Begum Ali, Gossé et al., in review; Keating et al., 2021; MacDuffie et al., 2020) on the same time course as other brain changes (e.g., Elsabbagh & Johnson, 2010; Gui et al., 2021; Piven et al., 2017).

Therefore, it is important to understand the interrelation between sleep and brain development in infancy and in particular whether individual differences in infant sleep are related to variation in early brain development.

Infant brain function can be assessed using electroencephalography (EEG), a method that is scalable, noninvasive, and suitable for use during more naturalistic capture of brain activity. Furthermore, EEG allows for the measurement of brain activity in complex, social contexts, where most early learning occurs (e.g., Dahl, 2015; Wass et al., 2018). Ongoing EEG can be decomposed into ongoing oscillations of different underlying frequencies that change with age. In general, power at frequencies in the lower range (i.e., theta; 3–6 Hz) decreases with age, whereas power in higher frequency bands increases with age (Anderson & Perone, 2018). Interestingly, this decrease in lower frequencies has been tentatively linked to synaptic pruning during development (Whitford et al., 2007). Apart from reflecting general patterns of brain maturation, oscillatory EEG activity has been linked to cognition/cognitive performance. In general, higher frequency oscillations are said to be important for local integration of information, whereas slower frequency oscillations are crucial for a more global integration of information (Buzsáki & Draguhn, 2004). Thus, examining EEG oscillations can provide markers of infant brain and cognitive development.

In the context of sleep, theta oscillations are a particularly important oscillatory frequency to study. A number of studies have shown that sleep propensity (the need to sleep) is related to waking (task‐related) theta power, with higher sleep propensity being associated with higher theta power. For example, Cajochen and colleagues (1995) showed that EEG power density increased in the 6–9 Hz range (considered to reflect theta frequency in adults) with prolonged (up to 36 hours) wakefulness in adults (Cajochen et al., 1995; see also Aeschbach et al., 1997; Finelli et al., 2000). This suggests that theta oscillations in adults are affected by sleep propensity. Indeed, increased frontal theta power has also been linked to subjective reporting of the “feeling of sleepiness” (Strijkstra et al., 2003). In children, one study showed a 45% increase of theta activity with the accumulation of sleep propensity during the day in 8‐ to 12‐year‐old children, particularly in frontal and central regions (Fattinger et al., 2017). Individual differences in habitual sleep during childhood also relate to daytime theta power: in one study, typically developing children with sleep problems (as measured by the parent‐reported Child Habit Sleep Questionnaire; Owens et al., 2000) had higher theta power than children without sleep problems (Winkelman et al., 2018). One view is that theta power represents a homeostatic marker of sleep (drive) (Borbély et al., 1989, Process S), that is, theta power increases with need for sleep during the day.

Theta oscillations have also recently attracted considerable interest as a potential unifying marker for general information processing in infancy (Jones et al., 2020; Meyer et al., 2019). Theta rhythms are thought to be generated in the hippocampus and facilitate information transfer between different brain structures (Buzsáki, 2009). Increases in frontal theta power during exploration have been associated with encoding of object memory (Begus et al., 2015), toy exploration (Orekhova et al., 2006), and language processing (Zhang et al., 2011) and are predictive of learning information (Begus & Bonawitz, 2020). Moreover, individual differences in the dynamic modulation of frontal theta in infancy (change over the course of a novel video) have been associated with later cognitive functioning across multiple cohorts (Braithwaite et al., 2020; Jones et al., 2020). Thus, examining the modulation of theta power in response to new information may provide insight into brain and cognitive development in infancy.

Despite the link between sleep and theta power in the adult brain, and the literature implicating individual differences in theta rhythms in infancy in cognitive development, there are few studies examining how individual differences in infant sleep relate to waking EEG theta rhythms. Friedrich and colleagues have examined in a series of papers the acute relationship between infant sleep and sleep EEG markers of development (Friedrich et al., 2015, 2017, 2019). They found that napping following learning benefits (semantic) memory consolidation as measured by ERP markers (e.g., N400 component) of memory. However, there are no studies (to the best of our knowledge) that examine oscillatory EEG markers of development in association with *habitual* infant sleep. Habitual infant sleep refers to an estimation of how infants usually sleep, rather than a one‐time study of the immediate effects of a nap. Studies of habitual sleep can be used to generate hypotheses as to the potential consequences of good and poor sleep for infant brain development.

Habitual infant sleep can be measured in several ways. Traditionally, infant sleep is measured by having parents fill out sleep questionnaires (e.g., Sleep and Settle Questionnaire [Matthey, 2001] and Brief Infant Sleep Questionnaire [BISQ] [Sadeh, 2004]) that enquire about an infant's sleep in the previous 1–2 weeks. Information about duration of sleep, night waking behaviors, and sometimes parental perception of the infant's sleep is recorded. Another method is sleep diaries, whereby parents are instructed to record sleep and wake times in infants for up to 10 days. Recently, actigraphy has gained popularity as an objective way to measure infant sleep patterns using accelerometer‐based estimations of sleep–wake behaviors in infants. Recent studies (Camerota et al., 2018; Gossé et al., 2022) have shown differing agreement between objective and subjective/parent report and in the association of subjective and objective sleep measures with markers of development (Gossé et al., 2022). For example, differences emerge between subjective and objective measures of night awakenings (e.g., Gossé et al., 2022; Sadeh, 1994; Tikotzky & Volkovich, 2019); of note, night awakenings that are captured by parental subjective reports are signaled night awakenings that require settling by parents, while actigraphy‐based measures are objective but may also capture briefer night awakenings that do not require help with settling. Thus, it is important to use comprehensive multi‐method assessments of sleep when examining relations to other aspects of development.

### 1.1 Present study

In summary, studies with children and adults have found that increased waking theta can track sleep propensity (Fattinger et al., 2017; Winkelman et al., 2018). Further, greater dynamic change in theta from baseline has been associated with both immediate learning, and later long‐term individual differences in learning and memory in infancy (Braithwaite et al., 2020; Jones et al., 2020). We propose that ongoing theta increases with sleep propensity, compromising the dynamic range that is achievable in response to novel content and consequently compromising learning and memory. To test this idea, we use a longitudinal design with multimodal sleep assessment to measure how habitual infant sleep patterns relate to both baseline theta power and dynamic changes in the theta rhythm in response to novel audiovisual material. We are including both objective and subjective measures of sleep to capture both sleep patterns (e.g. wakings) that are apparent to parents but also variation in sleep that is not directly captured by parents. We hypothesized that better infant sleep, for example, longer sleep duration/less fragmentation, would be associated with less baseline theta power, especially in frontal electrodes. Secondly, we hypothesized that better infant sleep would be associated with increased change in theta in response to presentation of a novel video (Braithwaite et al, 2020; Jones et al., 2020). Furthermore, we investigated change across infancy using 2‐month age windows. We expected absolute theta power to decrease with age and theta change in response to novel videos to increase with age as sleep duration increases and night waking decreases. We expect individual differences in sleep to have a larger effect for older infants as sleep is becoming more consolidated and variability in sleep decreases over time (Bathory & Tomopoulos, 2017).

## 2 METHODS

### 2.1 Participants

The present sample included 76 typically developing, term‐born (Gestational age (GA) > 37 weeks) infants (42 female) without a (familial) medical history of sleep or neurodevelopmental or neuropsychiatric disorders assessed by parent report. The age range at recruitment ranged from 4 to 14 months (mean age: 282 days, *SD* = 92 days, range in days: 116–456 days). Exclusion criteria were a family history of neurodevelopmental and/or sleep disorders, premature birth (< 37 GA), and plans to move away from the Frankfurt area in the near future. Section 1 in the Supporting Information shows details of sample size at each visit including mean age at each visit.

### 2.2 Design

**FIGURE 1:**
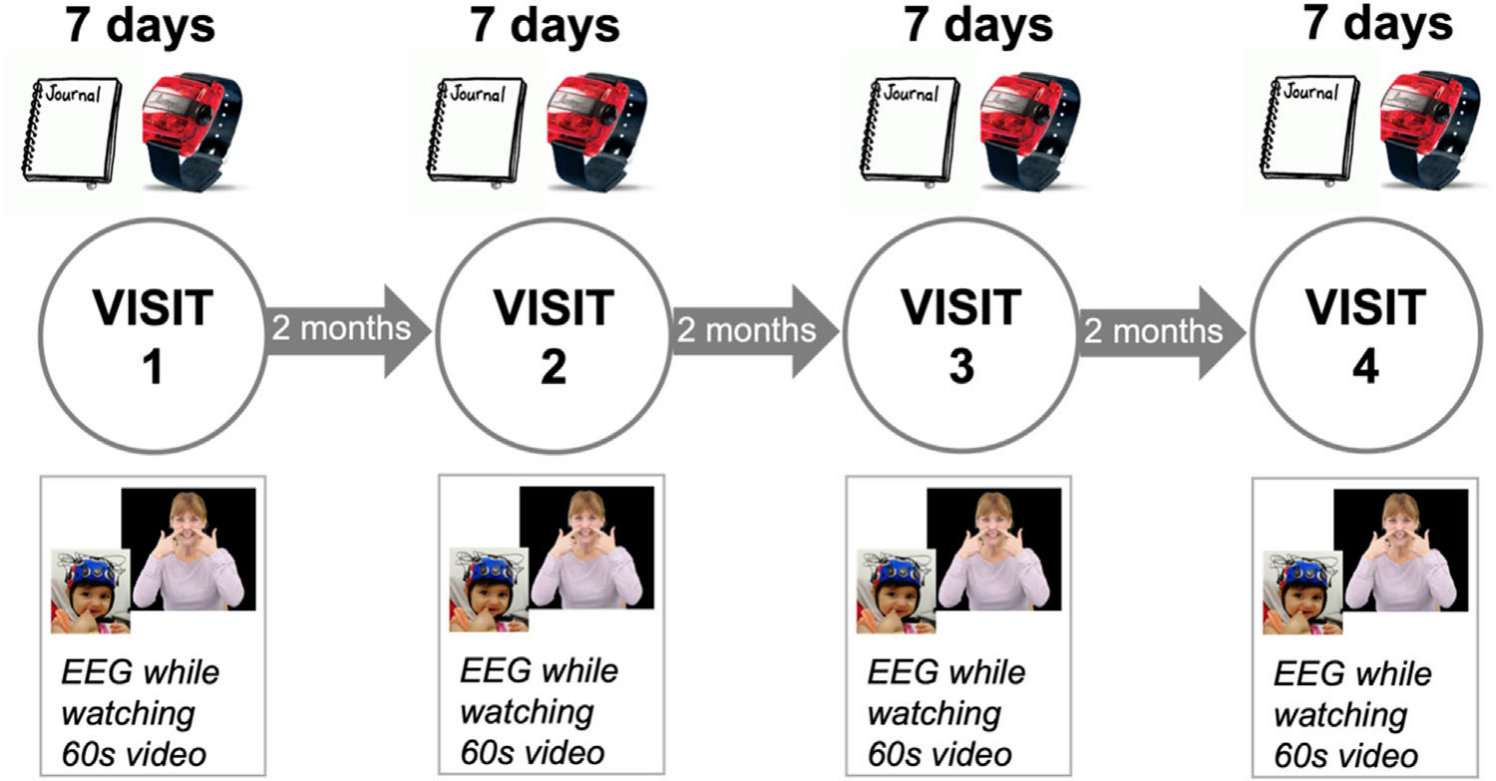
Study design

The design of this study is an accelerated longitudinal design to be able to collect more data from more infants in the limited project timeline. Infants entered the study at age brackets 4, 6, 8, 10, and 12 months of age and were followed every 2 months until they reached 14 months or had completed four visits. Cross‐sectional data were also collected at 14 months to increase the sample size at that age. All analyses included all age ranges. See Figure 1 for an illustration of the study design.

### 2.3 Sleep measures

#### 2.3.1 Sleep diary

Parents completed an in‐house sleep diary every day for a week at each time point. For details, see Gossé et al. (2022). The sleep diary enquired about an infant's bedtime routines (e.g., lullabies, story reading, or feeding at bedtime), bedtime and wake up times, night wakings, naps, and nap routines. Sleep duration markers and night waking information were calculated per night and day and then averaged for the week to obtain estimations of night and day sleep duration, night waking number, and night waking duration (WASO). A copy of the sleep diary can be found in Section 6 in the Supporting Information.

#### 2.3.2 Actigraphy

We used the ActiGraph wGT3X‐BT from ActiGraph Corp. (low‐frequency band pass filter: 0.25−2.50 Hz), which has been used in previous work (Barreira et al., 2018; Nyström et al., 2017; Reddy et al., 2018). The actigraph was set to zero‐crossing mode. Caregivers were encouraged to let the infant wear the actigraph for 24 h for 7 days and asked to record duration and dates for when the actigraph was removed. These periods were later used during analysis to exclude relevant periods (in addition to the ActiLife Software algorithm). Based on literature (Meltzer et al., 2012; Pisch, 2015), sampling frequency for our device was 60 Hz and at least five 24‐h segments of data were required for the dataset to qualify for inclusion in the final actigraphy dataset. Strategies to improve compliance included instruction sheets for use of the actigraph and personal communication with parents during the study week. The Sadeh algorithm was used to score sleep–wake data (Sadeh et al., 1991). Similar to the diary data, sleep duration markers and night waking information were calculated for actigraphy per night and day and then averaged for the week to obtain estimations of night and day sleep duration, night waking number, and night waking duration.

#### 2.3.3 Brief Infant Sleep Questionnaire

The BISQ is a short, standardized screening questionnaire that assesses habitual infant sleep patterns in the week prior to completion date. BISQ completion time is 5–10 min. Questions enquire about classical sleep parameters such as sleep duration or number of night waking but also about parental perception of sleep and sleep routines. Good validity was established by Sadeh (2004), who compared BISQ scores to actigraphy and sleep diaries. Test–retest reliability ranged from .82 to .95 (Sadeh, 2004). Lewandowski et al. (2011) found moderate correlation of BISQ and caregiver reports but stated that the BISQ was sensitive enough to track developmental sleep trends (Lewandowski et al., 2011). We extracted the same sleep measures for actigraphy and diary: sleep duration (day and night), night waking duration (WASO), and night waking number.

### 2.4 EEG measures

#### 2.4.1 EEG system

The EEG system used in this study was a 20‐channel, wireless Enobio System (Neuroelectrics, BCN, ES). Gel‐based electrodes are embedded in a soft neoprene cap and connected to a Bluetooth transmitter. The sampling frequency was 500 Hz. References were placed on the right mastoid. The EEG setup covered the whole head, according to the 10–20 system.

#### 2.4.2 EEG task

We used EEG data collected during the presentation of two 1‐min videos of women singing different nursery rhymes, with corresponding hand gestures, in an unfamiliar language (Swedish). Social videos were chosen as they had elicited the strongest theta response in prior studies compared to other videos (Jones et al., 2015). The videos were interspersed with other stimuli within an EEG testing battery that lasted 15–20 min.

### 2.5 Analysis plan

#### 2.5.1 EEG preprocessing

**FIGURE 2:**
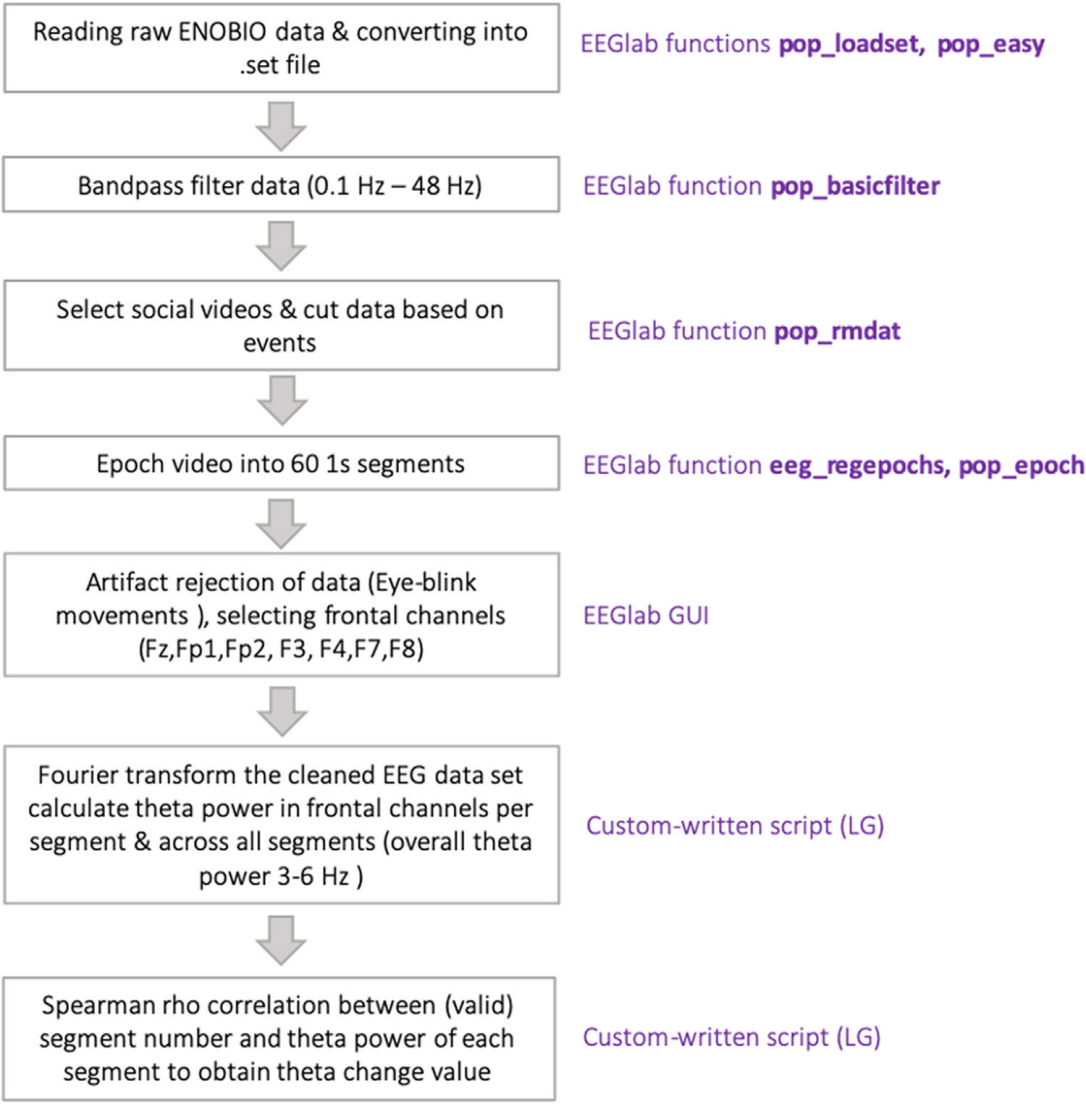
Illustration of EEG preprocessing pipeline.

EEG data preprocessing was done using EEGlab version 19.1 and custom‐written MATLAB scripts using Matlab version 2019a. Details of the preprocessing steps can be found in Figure 2. Data were bandpass filtered at 0.1–48 Hz. Data were segmented into 60 1‐second epochs for each video and artifact rejection was performed by manual rejection. Datasets were only included if at least 15 epochs per each half of the social video were valid (Braithwaite et al., 2020). Bad channels were excluded on a subject‐by‐subject basis, but at least four frontal channels were required for inclusion of the dataset. Only the first social video was used for theta change analysis and the first and second videos for theta power calculation to maximize the amount of complete data. After segmentation and artifact rejection, the datasets were Fourier transformed (Matlab 2019a function fft) to obtain theta power (defined as 3–6 Hz, as in Braithwaite et al., 2020; Jones et al., 2020) of each 1‐s segment as well as across the entire number of valid segments of the video. Overall theta power was obtained by averaging theta power across all valid segments of the two social videos across all available frontal channels. To calculate theta power change, the theta power value of each segment was correlated with the segment numbers within the first video and Spearman's rho was extracted as the dependent variable (see Braithwaite et al. [2020], Index b).

**FIGURE 3:**
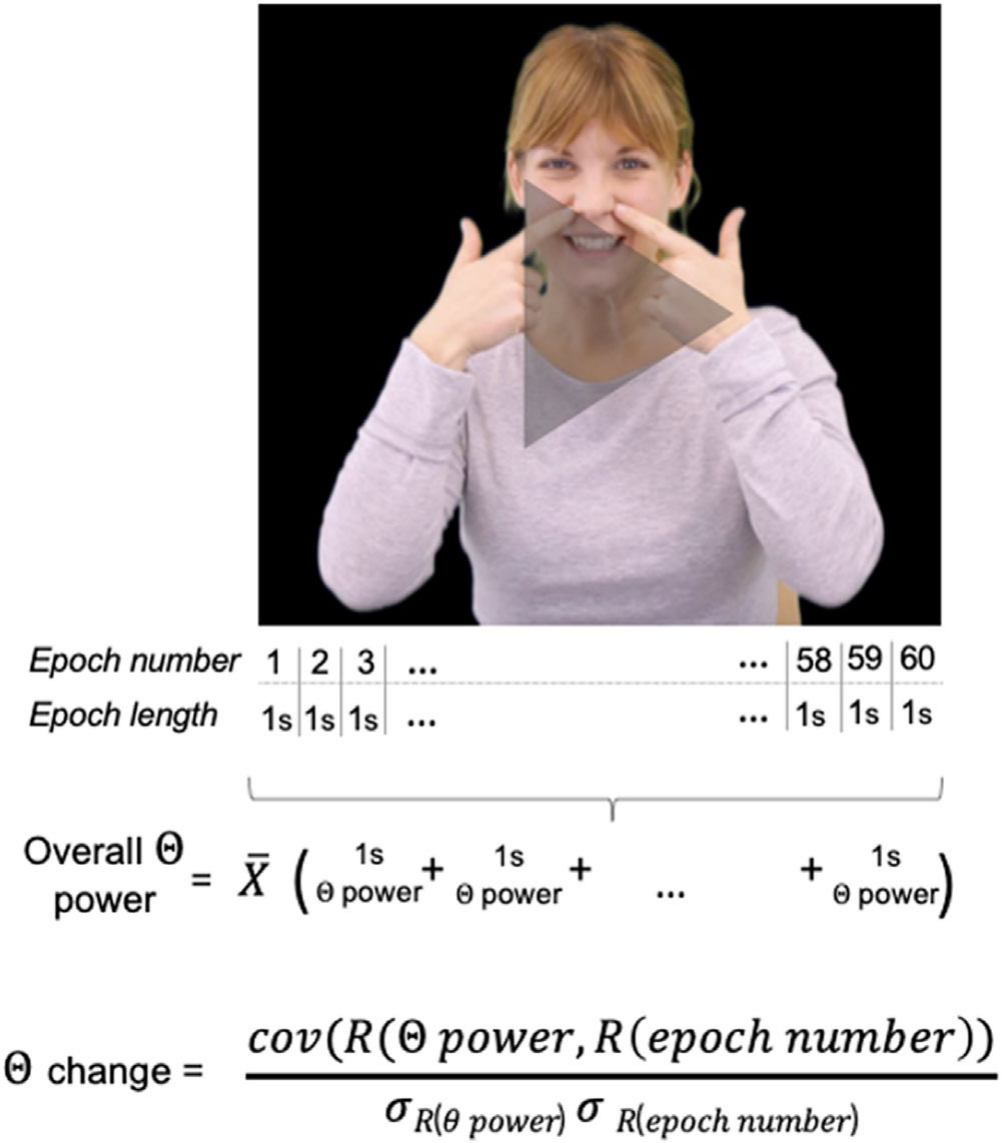
Illustration of a definition of theta power versus theta change. Overall theta power was calculated across the first two videos and theta change within the first video only.

Figure 3 illustrates how theta power versus theta change were calculated.

#### 2.5.2 Data exclusion

We could not collect EEG data from *N* = 25 sessions due to infant fussiness or parents not feeling comfortable with the EEG. We excluded *N* = 30 sessions due to noisy data (Heartrate (HR), electrode pops, eye movement artifacts, etc.). Of note, HR artifacts were prominent in younger populations as mastoid reference placement was harder due to limited space. We excluded *N* = 15 sessions due to experimenter/equipment error/event file error, which occurred primarily in the beginning of testing for this study. The final sample was 84 datasets from 60 infants across four visits.

#### 2.5.3 Statistical analysis

Linear mixed‐effects modelling (LMMs) was used to analyze the data because some children contributed data at more than one timepoint. For details regarding different models tested and rationale for analysis choice, see Section 2 in the Supporting Information. Below, the full LMMs tested are specified. Overall theta power and theta power change were the dependent variables. The full LMMs tested are as followed. A baseline model assessed how the theta parameters changed over time. Model 1 describes a model where main effects of age group and sleep variables were tested; Model 2 added an interaction effect of age group by sleep variable; and Model 3 added gender. For details on actigraphy preprocessing, see Gossé et al. (2022).

## 3 RESULTS

### 3.1 Descriptive statistics for theta power and theta change

Table S2 shows the descriptive statistics for theta power and theta power change. An illustration of the power spectrum per age bracket can be found in Section 4 in the Supporting Information.

### 3.2 Developmental changes in theta power and theta change

**FIGURE 4:**
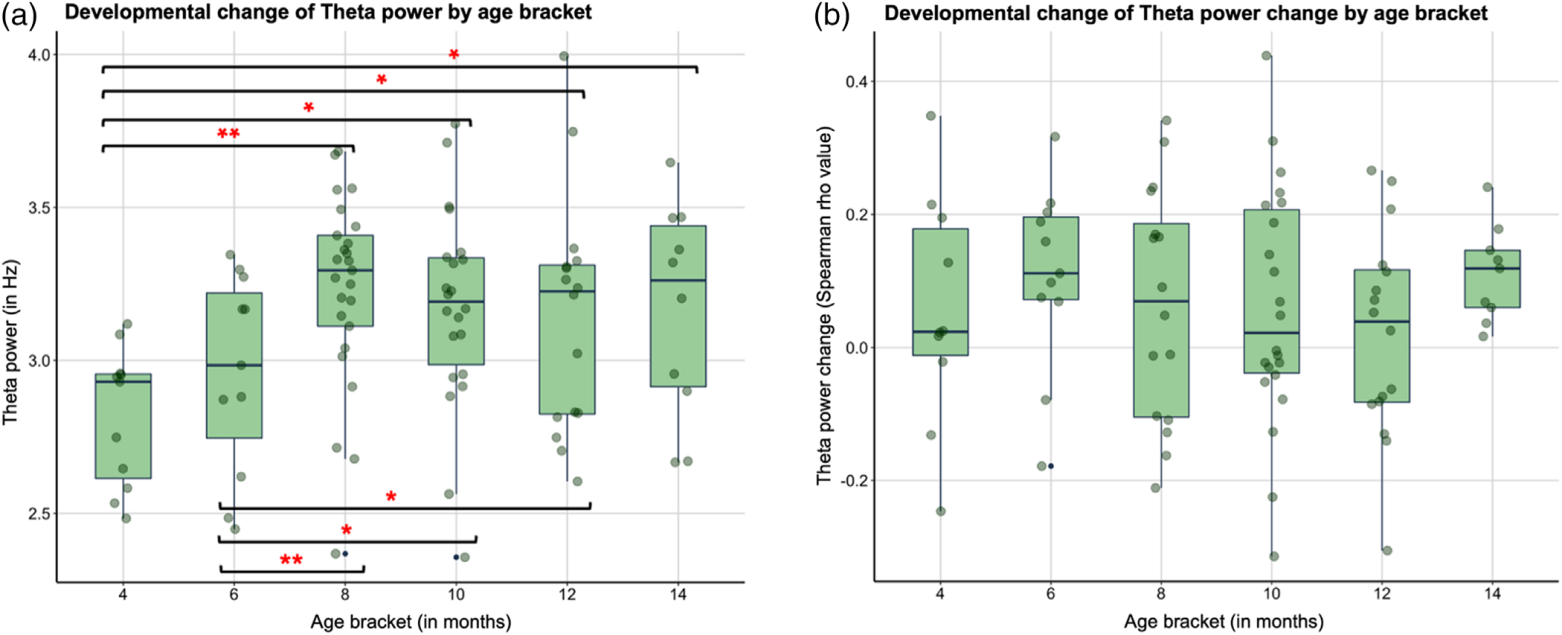
(a) Developmental change of theta power. (b) Developmental change of theta power change. *p < .05; **p < .001

Developmental changes were only observed in overall theta power (*F*(5,57) = 7.61, *p* < .001) but not with theta power change (*F*(5,73) = 0.63, *p* = .67). Contrary to our prediction, theta power increased with age; follow‐up analyses for overall theta power showed that age bracket differences were significant between 4 and 8 (Mean difference (*MD*) = −0.48, *p* < .001), 4 and 10 (*MD* = −0.41, *p* = .003), 4 and 12 (*MD* = −0.46, *p* = .002), and 4 and 14 (*MD* = −0.42, *p* = .03) months of age. Age bracket differences were also significant between 6 and 8 (*MD* = −0.39, *p* = .001), 6 and 10 (*MD* = −0.32, *p* = .01), and 6 and 12 (*MD* = −0.37, *p* = .01) months of age. Multiple comparison correction was conducted using the Bonferroni method. For detailed statistical results, refer to Section 4 in the Supporting Information, and for illustration, see Figure 4a,b.

### 3.3 Sleep parameters and overall theta power and theta power change

Below, the results of the LMMs of sleep parameters in association with theta power (change) are briefly described. Detailed statistical results can be found in the Supporting Information. First, we describe the results of objectively measured sleep parameters in association with theta power (change). We then examined subjectively measured sleep parameters with theta power (change) to identify relations that were consistent across both objective and subjective measures.

#### 3.3.1 The association of theta power with objectively measured sleep

**FIGURE 5:**
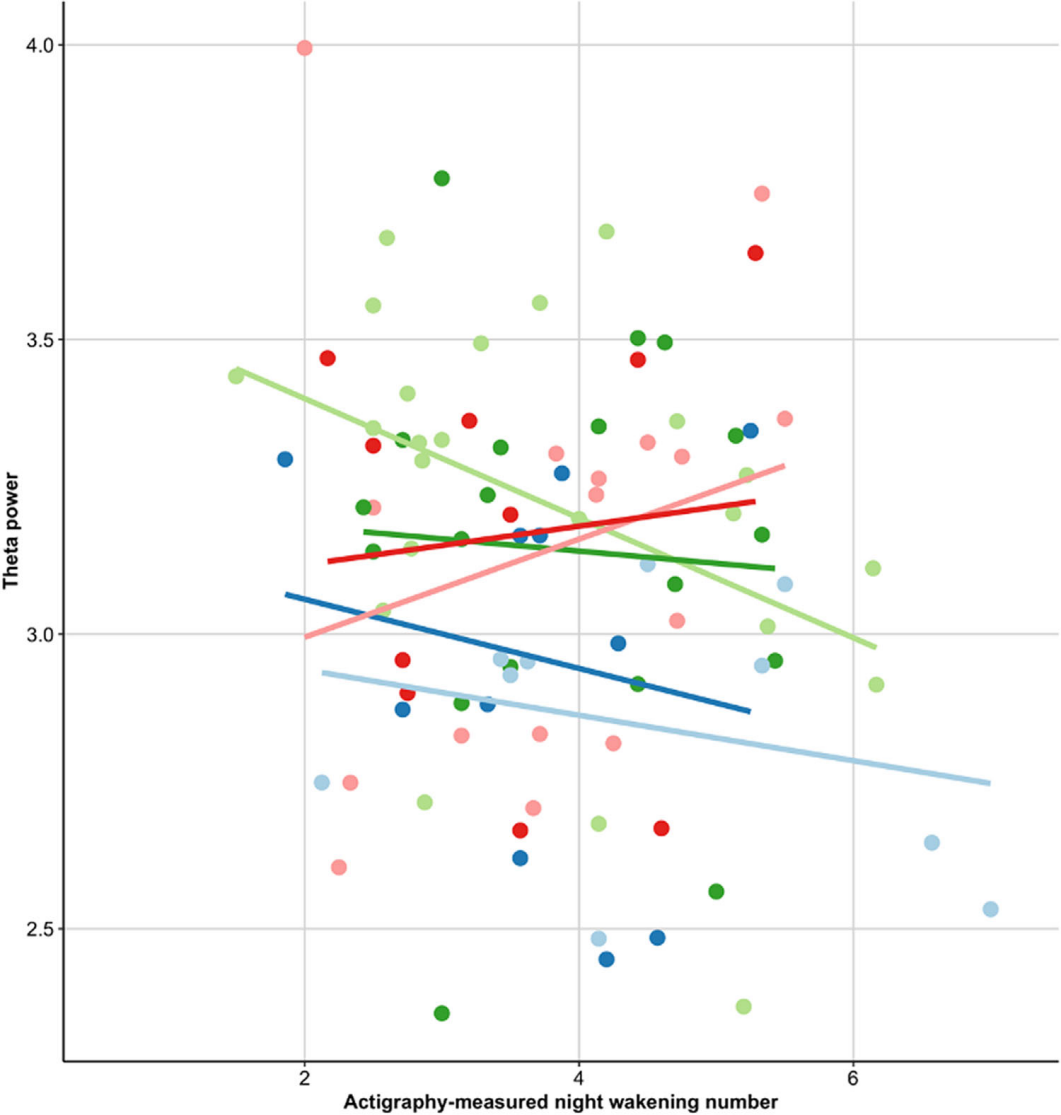
Developmental association of night waking number and theta power—Actigraphy. Developmental changes in the relationship between objectively measured night waking and overall theta power.

There was a significant interaction between the association between theta power and actigraphy‐measured night waking number and age group, such that in younger infants more night waking is related to lower overall theta power, and in older infants the relation was reversed (*F*(5,21) = 4.24, *p* = .008). Bonferroni‐corrected post hoc analyses showed significant differences between 4 and 6 months of age and 8, 10, 12, and 14 months of age (all *p*s < .001) but not within these brackets. For illustration, see Figure 5. None of the other sleep parameters (day, night, and wake after sleep onset duration) showed significant association with overall theta power (all *p*s > .05; see the Supporting Information).

#### 3.3.2 The association of theta power with subjectively measured sleep

Again, the association between theta power and diary‐measured night waking number varied by age bracket (*F*(5,27) = 3.25, *p* = .02) such that in younger infants more night waking related to lower overall theta power, and in older infants the relation was reversed. Diary‐measured developmental changes were similar to the actigraphy effects described above, though not identical. Post hoc analyses (Bonferroni corrected) showed significant differences between 4 and 6 months of age and 8, 10, 12, and 14 months of age (all *p*s < .001) but not within these brackets. There was a significant effect of BISQ‐measured night waking number (*F*(1,59) = 10.32, *p* = .002; best model = M3); however, this did not survive the removal of an outlier (*F*(1,31) = 0.45, *p* = 0.51; best model = M1) and therefore no post hoc analyses were conducted. This indicates that caution is warranted in the interpretation of the BISQ finding.

Though actigraphy data did not show associations of overall theta power with other sleep parameters (day and night sleep duration and WASO), we investigated these associations within the subjective measures for completeness, nonetheless. However, we did not find any additional associations (all *p*s > .05; see the Supporting Information for details).

#### 3.3.3 The association of theta change with objectively measured sleep

**FIGURE 6:**
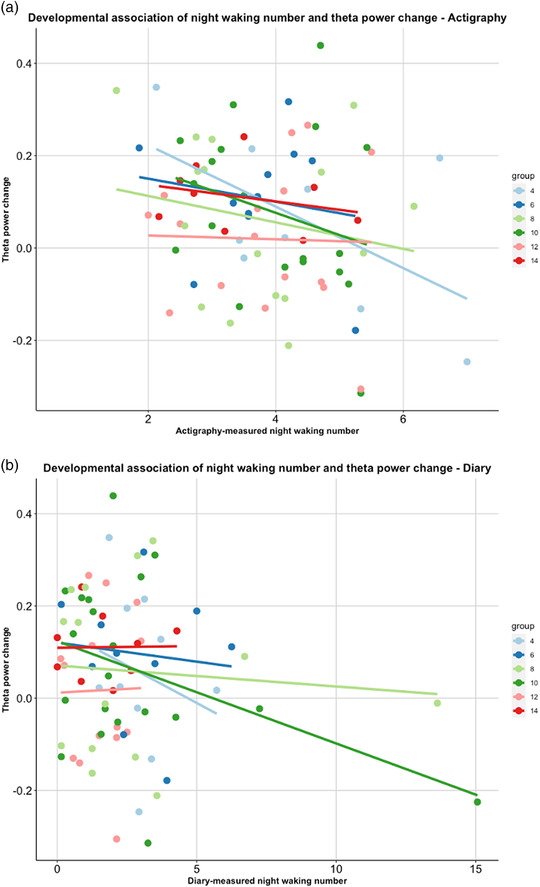
Illustration of the negative relationship between night waking number (objective and subjective sleep measures) and theta change

There was a significant association between theta change and actigraphy‐measured night waking number such that theta change decreased with increased night waking (*F*(1,65) = 5.49, *p* = .02). For illustration, see Figure 6. None of the other objectively measured sleep parameters (day and night sleep duration and WASO) showed significant association with theta change (all *p*s > .05; see the Supporting Information).

#### 3.3.4 The association of theta change with subjectively measured sleep

Similarly, there was a significant association between theta change within the video and diary‐measured night waking number (*F*(1,76) = 4.37, *p* = .04) such that theta change decreased with increased night waking; for BISQ, this was not significant.

Though, as above, actigraphy data did not show associations of theta change with other sleep parameters (day and night sleep duration and WASO), we investigated these associations within the subjective measures for completeness, nonetheless. There was no significant relationship between diary‐measured WASO and theta power change and a significant effect of BISQ‐measured WASO (*F*(1,79) = 4.40, *p* = .04), with increased WASO being associated with lower theta change.

## 4 DISCUSSION

We investigated the impact of sleep on overall theta power and theta power change in a longitudinal study. EEG data were collected from 4‐ to 14‐month‐old infants while they watched naturalistic social videos. Overall theta power in response to watching the social video and theta power change across the video was measured. Habitual sleep was measured using both objective and subjective methods over a week prior to the EEG visit. We hypothesized that better infant sleep (i.e., as indicated by longer sleep duration/less fragmentation) would be associated with less overall theta power while infants viewed social stimuli. Secondly, we hypothesized that better infant sleep (longer sleep duration/less fragmentation) would be associated with higher theta change values. Results indicated that greater sleep fragmentation was associated with higher theta power in older infants, but lower theta power in younger infants, indicating important effects of age in the relation between sleep and brain function. As predicted, across age groups greater sleep fragmentation was related to less modulation of theta during the social videos. Taken together, this is partially consistent with our proposal that poor sleep may raise daytime theta and reduced dynamic change in infancy.

### 4.1 Overall theta power in relation to sleep

Theta power increased with age, though this change was only significant between 4 months and the older age groups and between 6 months and the older age groups. Changes were not significant between 8 and 14 months of age, such that the maturational trajectory of theta power leveled off after 8 months. These changes could reflect brain maturation in the first year of life, which includes pruned synapses and white and gray matter changes (Anderson & Perone, 2018; Whitford et al., 2007). Previous research on theta across development showed a general decrease in the power spectral density of the theta band from 5 months to 2 years (Marshall et al., 2002) and a decrease in baseline theta power in 8‐ to 18‐year‐old children (Liu et al., 2014). In addition to maturational changes, individual differences in theta power might reflect cognitive differences. For example, research in infancy (6–12 months) showed that lower overall theta power was associated with lower levels of attention (Perone & Gartstein, 2019). As theta has been proposed to be related to attention (Michel et al., 2015), increases in overall theta observed in the present cohort may reflect the underlying development of attentional abilities. It is possible that our data reflect different mechanisms impacting theta at the same time, that is, while attentiveness to the videos increases with age might drive increased theta, less daytime sleepiness might decrease it. Future studies should track theta power across different times of day in infants to disentangle which factor drives individual theta power.

Recent research into the theta social brain network shows an emergence and a shift toward frontal brain regions of this network from 6 to 12 months of age (van der Velde et al., 2021); it is possible that the social nature of our stimuli drives some of the maturational findings. See further discussion below.

Sleep research has shown that theta power increases with sleep deprivation in adults (Cajochen et al., 1995); thus, we hypothesized an association between theta power and night sleep. Specifically, we predicted that poorer night sleep would be related to higher theta power in infants, suggesting a perpetual state of latent sleep deprivation. Our findings indicate that infant habitual sleep *duration* does not relate to overall wake theta power. However, all three sleep measures show that sleep *fragmentation* (operationalized as the number of night wakings) predicted overall theta power. Further, there were developmental changes in the nature of this relationship. Specifically, greater actigraphy‐measured night waking number was associated with lower theta power in the younger age groups but with higher theta power at 12 and 14 months, the latter age groups being in line with our hypothesis.

For the diary, we observed a similar pattern. Cross‐method differences between sleep methods in terms of results may be explained by inherent cross‐method differences in sleep measures (Gossé et al., 2022; Horger, Marsiliani, et al., 2021). We previously found in this cohort that mothers with higher stress and with infants who were older were more likely to show consistency in subjective and objective sleep measures (Gossé et al., 2022); it is thus interesting that both diary and actigraphy show similar associations. Robust associations between sleep and neurocognitive measures should be captured by different sleep assessment methods regardless of underlying influences on cross‐method agreement. Our results may suggest that signaled wakings have the strongest relation to daytime theta, because these are represented in both the objective and subjective measures. The ability to self‐regulate is broadly linked to attention and executive functioning in infancy (Hendry et al., 2016; Ursache et al., 2013). Potentially, younger infants who have a higher number of signaled wakings have less well‐developed self‐regulation skills (which may be adaptive, given the need for more frequent feeding), and this may be reflected in lower levels of daytime theta power. Supportive of this is research showing that theta oscillations are linked to the development of sustained attention (Xie et al., 2018), which in turn has been linked to self‐regulation abilities (Brandes‐Aitken et al., 2019). Toward the end of the first year of life, infants learn how to self‐regulate and go back to sleep by themselves; infants who continue to wake may then show higher daytime theta because of sleep propensity.

Night waking number (or sleep fragmentation, as called in the following) is sometimes taken as an indicator for poor sleep (Pennestri et al., 2018). Fragmented sleep is not as restorative as uninterrupted sleep. This may lead to more daytime sleepiness and, thus, increased theta overall. Sleep fragmentation has been linked to less slow wave sleep (Bonnet, 1985), which is crucial for memory formation and synaptic plasticity and therefore learning (Tononi & Cirelli, 2003, 2014). The pattern seen in the older infants (of increased wakings relating to higher theta power) is thus consistent with the idea that theta power is a marker of sleep propensity. Interestingly, adult insomniacs (a disorder characterized by poor, often fragmented, sleep) exhibited lower theta power during wakeful periods, which the authors interpreted as lower sleep drive (Wołyńczyk‐Gmaj & Szelenberger, 2011). We think this could potentially explain the pattern we see in younger infants. Early sleep propensity systems are less mature and therefore we might get a different relationship to daytime theta.

Of course, as we conducted correlational analyses, we cannot assume causality in the relationships we found. It is possible that both theta power and sleep duration are markers of general maturity. As such, excess theta power could be a marker for slower brain maturation. Higher sleep fragmentation is common in younger infants and a sign of more immature sleep consolidation (Bathory & Tomopoulos, 2017). It could be that underlying immature brain anatomy affects both sleep and daytime theta power (Michels et al., 2011). Potentially consistent with this interpretation of theta power are findings that show that theta power is higher in children that suffered adversity (Marshall & Fox, 2004) and in children with epilepsy (Michels et al., 2011) compared to controls. However, this interpretation does not match the observed overall age‐related changes within the present study, whereby theta power increased with age (possibly due to the social nature of the stimulus).

### 4.2 Dynamic modulation of EEG theta and sleep

Dynamic increases in theta power within a stimulus may reflect increases in engagement and cognitive effort (Liu et al., 2014). Taken with previous research by Jones et al. (2020) and Braithwaite et al. (2020), where greater dynamic theta change within a novel video was found to predict individual nonverbal, cognitive ability, this may suggest that theta change reflects individual differences in processes such as attention, learning, and memory that contribute to cognitive growth.

Interestingly, across all three sleep measures fewer night wakings predicted greater change in theta power within a video. Prior research has shown that less theta change is indicative and/or predictive of poorer cognitive abilities (Jones et al., 2020; Braithwaite et al., 2020). This matches research that links sleep fragmentation to poorer performance on cognitive tasks (e.g., Pisch et al., 2019). Theta change in response to stimuli has been linked to task learning and memory (e.g., Meyer et al., 2019). Thus, our findings could indicate a broad link between fragmented sleep in infancy and later cognitive development; this is supported by prospective links between disrupted night sleep at 14 months and cognitive development at age 3 years (Begum‐Ali, Gosse et al., in review).

Associations with broader cognition may not emerge until later in development. Some studies (e.g., Mäkelä et al., 2018) have observed no associations between night waking and general cognitive function to age 24 months, or have observed specific relations to skills such as executive functioning (Bernier et al., 2013) or social competence (Mäkelä et al., 2021). Of note, analyses of parent report data from the present cohort showed associations between night waking and gross motor skills, but not other subscales of the Ages and Stages Questionnaire (ASQ; Gossé et al., 2022) and for most of the subscales neither absolute theta power nor theta change was associated with concurrent ASQ scores (see the Supporting Information for details). It is possible that sleep fragmentation in infancy initially affects specific cognitive processes (such as attention) that have later cascading effects on broader cognitive development (Braithwaite et al., 2020; Jones et al., 2020). Relatedly, theta oscillations might be associated with the emerging development of executive functioning (Perone et al., 2018), a foundational capacity for cognitive ability. Sleep research has shown sleep fragmentation to impact concurrent and later executive functioning (Bernier et al., 2013; Mäkelä et al., 2020) potentially explaining also the maturational trajectory in our data. Studies showing that associations of sleep with aspects of cognition in infancy (e.g., attention) cascade differentially onto general cognitive development/ability in later years might support this (Begum‐Ali, Gossé et al., in review). Future work should test the hypothesis that links between fragmented night sleep in infancy and later cognitive development are mediated by altered executive attention.

Future research should focus on understanding whether this finding is associated with our use of social stimuli in the video or whether night waking might modulate responses to social stimuli in particular (such as suggested already by Sun et al., 2016; 2018). A recent study showed that infants and toddlers waking up more at night (more than three times) as measured by subjective parent report showed stronger attentional bias toward fearful faces than their peers with fewer (signaled) night awakenings (Mäkelä et al., 2021). Previous research has shown both social and nonsocial stimuli to evoke theta responses (e.g., Braithwaite et al., 2020; Jones et al., 2020, 2015). However, some evidence shows that social information is preferentially encoded during sleep compared to nonsocial information (Reeb‐Sutherland et al., 2011). Therefore, it may be possible that the effect we see is unique to social stimuli. Supportive of this is the abovementioned research on theta social brain networks that develop specificity to social stimuli in the second half of the first year of life (Van der Velde et al., 2021).

There were no associations of theta change with day or night sleep duration in any of the measures. Further, there was no association of theta change with actigraphy‐ or diary‐measured WASO. WASO measures the duration that the infant was awake at night in total, whereas night waking number measures the number of times the infant woke up. Possibly theta rhythms are most impacted by the number of disruptions to sleep rather than the duration of the disruption. By that metric, being awake once a night for 30 min would not be as detrimental for cognitive performance/development as waking up many times a night for 3 or 4 minutes at a time. This could be because many short night wakings might disrupt memory consolidation processes that occur during sleep (e.g., Tononi & Cirelli, 2003). Thus, from the present data it appears that sleep fragmentation, rather than duration, links to theta oscillations in the infant brain.

### 4.3 Integration of the findings and potential mechanistic explanations

In summary, we observed relationships between EEG theta power and change and one aspect of sleep across measures: sleep fragmentation. The underlying mechanism through which sleep might influence theta oscillations, and therefore cognition, could be explained by the restorative properties of (slow wave) sleep and via the synaptic homeostasis hypothesis (SHY; Tononi & Cirelli, 2003). The SHY proposes that slow wave sleep is needed for “resetting synapses” after a day of learning new information, a skill vital for infants. Interrupting slow wave sleep with many night wakings might disrupt this process.

Importantly, our findings suggest that sleep fragmentation could be a potentially important intervention target (Roberts et al., 2018). As prior findings show theta power differs in children with neurodevelopmental disorders and in infants at risk for neurodevelopmental disorders (Jones et al., 2020) and given theta power's role in general information processing, targeting waking theta via sleep intervention could be promising. Sleep fragmentation can be targeted with simple sleep interventions in order to reduce the frequency of wakings, including adjustments to a child's sleeping environment (temperature, noise levels, etc.). Future studies could potentially include an intervention targeting sleep fragmentation and examine its effects on waking theta power. Should theta power (and theta power change) be responsive to manipulations of night waking frequency, this would provide evidence for a causal role of night waking in wake theta power.

### 4.4 Limitations

While our accelerated longitudinal design allowed us to increase sample sizes in each group, it limited our ability to employ statistical techniques that can model temporal relationships. Future studies should focus on disentangling the causal roles of sleep on theta via intervention studies. One challenge is the lack of consensus as to what (frontal) theta power in infants represents, with roles suggested for sleep propensity, attention, language or information processing, or cognitive control. Future work should try and disentangle the role of theta during wake and its direct connection to subsequent infant sleep. One possible way to tackle this could be to measure theta power across different times of the day (similar to Fattinger et al. [2017]) to observe if infants show homeostatic accumulation of theta power similar to adults. Moreover, future studies should combine measuring theta power and theta power change during wake with polysomnography measurements to identify whether slow wave sleep differs depending on individual theta power, as found in prior literature of other age groups. Lastly, frontal theta power might differ in its association with sleep depending on the age of the infant as our actigraphy findings might suggest. Future research should explore the association between theta power and sleep across the first year of life more closely.

## 5 CONCLUSION

In conclusion, our results suggest that examining night sleep parameters, and in particular measures of sleep fragmentation, is important when examining the relationship between individual differences in sleep and infant brain development. Strengths of our work included a large sample size and a multimethod assessment of infant sleep. More night waking is related to greater modulation of theta power during a social video, a measure that has been previously linked to longer term differences in cognitive ability. Moreover, there were age‐related changes in the relationship between overall theta power and habitual night waking number, that is, greater habitual night waking number was associated with lower theta power for younger age groups but with greater theta power for older infants. We interpret this as night waking adding an additional strain on an infant's attentional and self‐regulation abilities, where younger infants are less able to cope with the additional strain than older infants. Importantly, these EEG results converged well across objective and subjective measures. This underscores the benefit of direct measures of brain activity when examining the effects of individual differences in sleep and suggests that sleep fragmentation may impact brain development in infancy.

## CONFLICTS OF INTEREST

L.G. has worked as a paid consultant for P&G on work unrelated to this manuscript. The other authors declare no conflict of interest.

## Supporting information

Supplementary Material

## Data Availability

The data that support the findings of this study are available from the corresponding author upon reasonable request.
