# Supplementary material for: Habitual night waking associates with dynamics of waking cortical theta power in infancy
Source: Dev Psychobiol. 2022 Nov 21;64(8):e22344. doi: 10.1002/dev.22344 (PMC9828365; doi:10.1002/dev.22344)
Supplement: Supplementary file 1 — Supplementary Material [file DEV-64-0-s001.docx]

**Supplementary material**

Habitual night waking associates with dynamics of waking cortical theta power in infancy

Louisa K. Gossé^1*^, Frank Wiesemann^2^, Clare E. Elwell^3^, Emily J.H. Jones^1^

^1^Centre for Brain and Cognitive Development, Birkbeck, University of London, London, United Kingdom

^2^Research & Development, Procter & Gamble, Schwalbach am Taunus,
Germany

^3^Department of Medical Physics and Biomedical Engineering, Biomedical Optics Research Laboratory, University College London, London, United Kingdom

**Section 1 – Sample size in cross-sectional vs longitudinal part of study**

Table SM1 describes sample size tested for each age bracket.

| *Table SM1.* Sample sizes tested at each visit | | | | | | |
| --- | --- | --- | --- | --- | --- | --- |
|  | **Age (in months) tested →** | | | | | |
| **Age**  **(in months) enrolled ↓** | **4** | **6** | **8** | **10** | **12** | **14** |
| **4** | 17 (9f) | 5 | 6 | 5 | - | - |
| **6** | - | 19 (10f) | 15 | 12 | 9 | - |
| **8** | - | - | 22 (11f) | 11 | 6 | 5 |
| **10** | - | - | - | 5 (2f) | 4 | 3 |
| **12** | - | - | - | - | 9 (7f) | 9 |
| **14** | - | - | - | - | - | 4(3f) |
| **Total N/ age group**  *Mean age in days* | 17  *131±8* | 24  *187±12* | 43  *247±10* | 33  *306±10* | 28  *370±12* | 21  *431±11* |

*Notes.* Final sample size for each cohort, as well number of girls for each age bracket. f = girls. Total N = 76 participants (42f).

**Section 2 – Details of Linear Mixed Model Analysis**

LMMs were chosen to account for participants dependence and inter-individual variability (Fields, 2005). A random intercept was added for participant and a repeated measures effect for age bracket as an indicator for time to account for the fact that some participants were tested more than once. Age bracket was selected as categorical predictor of developmental change to identify critical periods and fine-grained age-related changes in the relationship between sleep and development.

A mixed model analysis allowed us to leverage the longitudinal data collected and account for within subject variability rather than only between subject variability, thus increasing statistical power and accounting for individual differences in development.

Covariance structures were specified as autoregressive type 1 (AR1) for the repeated group effect, as AR1 covariance structure assumes that two measures taken close together in time from a participant are more likely to be correlated than two measures taken further apart in time (Field, 2005). Random intercept was given a covariance structure of variance components that assumes all random effects to be independent. Pairwise comparisons corrected for multiple comparisons using the Bonferroni were performed for age groups and for sleep clusters where applicable. Model fit statistics were compared between models to choose the best model. Here, the Akaike Information Index (AIC) was used for model comparison (smaller the better the model fit). This was done as the AIC considers the number of predictors when calculating model fit (Field, 2005).

**Section 3 – Descriptive statistics**

| *Table SM2. Descriptive statistics overall theta power and theta power change* | | | | |
| --- | --- | --- | --- | --- |
|  | Minimum | Maximum | Mean | SD |
| Theta power video 1  (in Hz) | 2.38 | 3.98 | 3.08 | 0.35 |
| Theta power video 2  (in Hz) | 2.33 | 4.06 | 3.16 | 0.37 |
| Overall theta power  (in Hz) | 2.36 | 4.00 | 3.12 | 0.35 |
| Theta power change video 1 (spearmans rho =) | -0.31 | 0.44 | 0.06 | 0.16 |
| Theta power change video 2 (spearmans rho =) | -0.17 | 0.49 | 0.11 | 0.16 |
| *Note*. SD = Standard deviation | | | | |

As a validation for whether theta oscillations in response to the videos in our sample behave as expected based on prior research, we tested if absolute theta power increased from video 1 to video 2. This is based on research by e.g., Orekhova et al., (1999) and Wass et al. (2018) that showed an expected increase in theta power with task duration. “T-tests showed absolute theta power significantly increased from video 1 to video 2 [t (56) = -3.13, p = .003]. There was no difference in within-video theta change between video 1 and 2 [t (57) = -1.52, p = .14].”

**Section 4 - Power spectrum of EEG by age bracket**

**
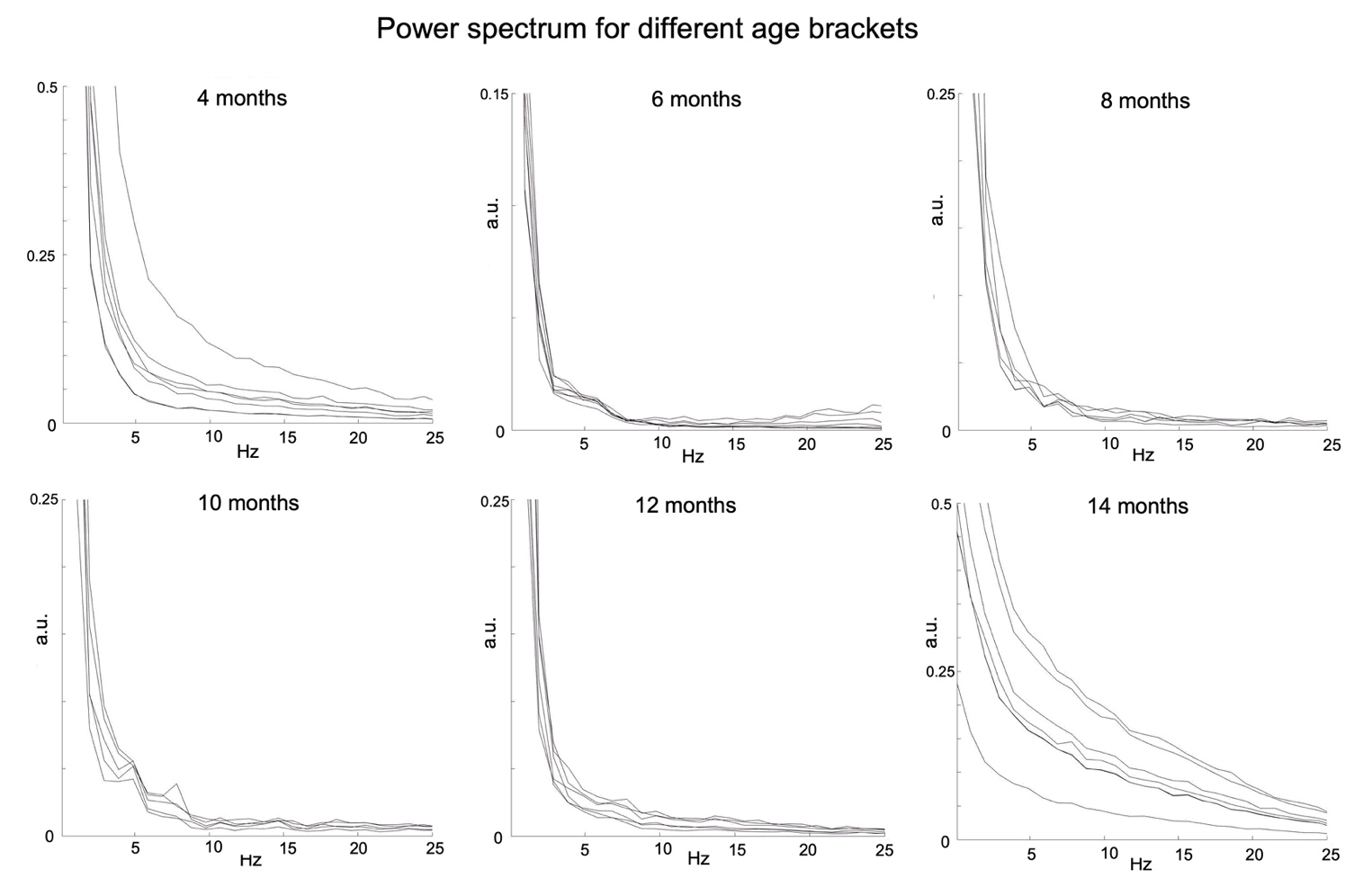
**

*Note.* Individual lines represent average power spectrum for the seven frontal channels*.*

**Section 5**- **Linear mixed models theta power – sleep parameters**

*Table SM3a-l* reports the statistical results and model fit statistics for the Linear mixed models run and that are presented in the main text.

| *Table SM3a.*  Actigraphy night waking number and EEG theta power | | | | |  |
| --- | --- | --- | --- | --- | --- |
|  |  | **AIC** | **BIC** | **-2LL** | **df** |
| Theta power | Baseline | 58.21 | 81.20 | 40.21 | 9 |
|  |  | *Age group:* *F*(5,57) = 7.61, *p* <.001** | | |  |
|  | M1 | 41.20 | 66.09 | 21.20 | 10 |
|  | M2 | 40.95 | 78.28 | 10.95 | 15 |
|  |  | *Age group: F*(5,51) = 9.62, *p* < .001** | | | |
|  |  | *Night waking number :* *F*(1,82) = 2.65, *p* = .11 | | | |
|  |  | *Age group x Night waking number : F*(5,21) = 4.24, *p* = .008* | | | |
|  | M3 | 41.42 | 81.24 | 9.42 | 11 |
| Theta power change (video 1) | Baseline | -57.48 | -35.60 | -75.48 | 9 |
|  |  | *Age group:* *F*(5,73) = 0.63, *p* < .68 | | |  |
|  | M1 | -57.87 | -34.05 | -77.87 | 10 |
|  |  | *Age group: F*(5,51) = 0.60, *p* = .70 | | |  |
|  |  | *Night waking number :* *F*(1,65) = 5.50, *p* = .02* | | |  |
|  | M2 | -43.87 | -9.11 | -73.87 | 15 |
|  | M3 | -49.60 | -24.11 | -71.60 | 11 |
| *Notes.* *p <.05, **p <.001, °did not survive multiple comparison correction with the Bonferroni method, AIC = Akaike’s Information Criterion, BIC = Schwarz’s Bayesian Criterion, -2LL = -2 Log Likelihood, df = degrees of freedom. | | | | | |

| *Table SM3b.* Actigraphy WASO and EEG theta power | | | | |  |
| --- | --- | --- | --- | --- | --- |
|  |  | **AIC** | **BIC** | **-2LL** | **df** |
| Theta power | Baseline | 58.21 | 81.20 | 40.21 | 9 |
|  |  | *Age group:* *F*(5,57) = 7.61, *p* <.001** | | |  |
|  | M1 | 44.04 | 69.04 | 24.04 | 10 |
|  |  | *Age group: F*(5,29) = 14.39, *p* <.001** | | | |
|  |  | *WASO:* *F*(1,67) = 0.11, *p* = .74 | | | |
|  | M2 | 47.80 | 85.29 | 17.80 | 15 |
|  | M3 | 44.03 | 71.53 | 22.03 | 11 |
| Theta power change (video 1) | Baseline | -57.48 | -35.60 | -75.48 | 9 |
|  |  | *Age group:* *F*(5,73) = 0.63, *p* < .68 | | |  |
|  | M1 | -54.72 | -30.78 | -74.72 | 10 |
|  |  | *Age group: F*(5,58) = 0.60, *p* = .70 | | |  |
|  |  | *WASO:* *F*(1,78) = 0.23, *p* = .63 | | |  |
|  | M2 | -46.44 | -10.53 | -76.44 | 15 |
|  | M3 | -52.85 | -26.51 | -74.85 | 11 |
| *Notes.* *p <.05, **p <.001, °did not survive multiple comparison correction with the Bonferroni method, AIC = Akaike’s Information Criterion, BIC = Schwarz’s Bayesian Criterion, -2LL = -2 Log Likelihood, df = degrees of freedom. | | | | | |

| *Table SM3c.* Actigraphy Night sleep duration and EEG theta power | | | | |  |
| --- | --- | --- | --- | --- | --- |
|  |  | **AIC** | **BIC** | **-2LL** | **df** |
| Theta power | Baseline | 58.21 | 81.20 | 40.21 | 9 |
|  |  | *Age group:* *F*(5,57) = 7.61, *p* <.001** | | |  |
|  | M1 | 43.61 | 68.49 | 23.61 | 10 |
|  |  | *Age group: F*(5,26) = 19.37, *p* <.001** | | | |
|  |  | *Night sleep duration:* *F*(1,71) = 1.73, *p* = .19 | | | |
|  | M2 | 49.10 | 86.43 | 19.10 | 15 |
|  | M3 | 43.85 | 71.23 | 21.85 | 11 |
| Theta power change (video 1) | Baseline | -57.48 | -35.60 | -75.48 | 9 |
|  |  | *Age group:* *F*(5,73) = 0.63, *p* < .68 | | |  |
|  | M1 | -53.03 | -29.21 | -73.03 | 10 |
|  |  | *Age group: F*(5,55) = 0.53, *p* = .76 | | |  |
|  |  | *Night sleep duration:* *F*(1,62) = 0.44, *p* = .51 | | |  |
|  | M2 | -45.94 | -10.21 | -75.94 | 15 |
|  | M3 | -51.21 | -25.01 | -73.21 | 11 |
| *Notes.* *p <.05, **p <.001, °did not survive multiple comparison correction with the Bonferroni method, AIC = Akaike’s Information Criterion, BIC = Schwarz’s Bayesian Criterion, -2LL = -2 Log Likelihood, df = degrees of freedom. | | | | | |
| *Table SM3d.* Actigraphy Day Sleep Duration and EEG theta power | | | | |  |
|  |  | **AIC** | **BIC** | **-2LL** | **df** |
| Theta power | Baseline | 58.21 | 81.20 | 40.21 | 9 |
|  |  | *Age group:* *F*(5,57) = 7.61, *p* <.001** | | |  |
|  | M1 | 44.16 | 68.59 | 24.16 | 10 |
|  | M2 | 48.05 | 84.67 | 18.05 | 15 |
|  | M3 | 44.13 | 71.00 | 22.13 | 11 |
|  |  | *Age group: F*(5,29) = 11.05, *p* <.001** | | | |
|  |  | *Day Sleep Duration:* *F*(1,73) = 1.67, *p* = .201 | | | |
|  |  | *Age group x Day Sleep Duration: F*(1,52) = 2.07, *p* = .16 | | | |
| Theta power change (video 1) | Baseline | -57.48 | -35.60 | -75.48 | 9 |
|  |  | *Age group:* *F*(5,73) = 0.63, *p* < .68 | | |  |
|  | M1 | -50.68 | -27.24 | -70.68 | 10 |
|  |  | *Age group: F*(5,55) = 0.62, *p* = .68 | | |  |
|  |  | *Day Sleep Duration:* *F*(1,76) = 0.61, *p* = .44 | | |  |
|  | M2 | -44.02 | -8.86 | -74.02 | 15 |
|  | M3 | -48.68 | -22.89 | -70.68 | 11 |
| *Notes.* *p <.05, **p <.001, °did not survive multiple comparison correction with the Bonferroni method, AIC = Akaike’s Information Criterion, BIC = Schwarz’s Bayesian Criterion, -2LL = -2 Log Likelihood, df = degrees of freedom. | | | | | |
| *Table SM3e.* Diary Night waking number and EEG theta power | | | | |  |
|  |  | **AIC** | **BIC** | **-2LL** | **df** |
| Theta power | Baseline | 58.21 | 81.20 | 40.21 | 9 |
|  |  | *Age group:* *F*(5,57) = 7.61, *p* <.001** | | |  |
|  | M1 | 41.82 | 66.48 | 21.82 | 10 |
|  | M2 | 39.59 | 76.58 | 9.59 | 15 |
|  |  | *Age group: F*(5,29) = 9.30, *p* <.001** | | | |
|  |  | *Night waking number :* *F*(1,65) = 6.62, *p* = .01* | | | |
|  |  | *Age group x Night waking number : F*(5,27) = 3.25, *p* =.02* | | | |
|  | M3 | 41.02 | 80.48 | 9.02 | 11 |
| Theta power change (video 1) | Baseline | -57.48 | -35.60 | -75.48 | 9 |
|  |  | *Age group:* *F*(5,73) = 0.63, *p* < .68 | | |  |
|  | M1 | -48.88 | -25.44 | -68.88 | 10 |
|  |  | *Age group: F*(5,49) = 0.53, *p* = .75 | | |  |
|  |  | *Night waking number :* *F*(1,76) = 4.37, *p* = .04* | | |  |
|  | M2 | -43.20 | -8.04 | -73.20 | 15 |
|  | M3 | -46.98 | -21.20 | -68.98 | 11 |
| *Notes.* *p <.05, **p <.001, °did not survive multiple comparison correction with the Bonferroni method, AIC = Akaike’s Information Criterion, BIC = Schwarz’s Bayesian Criterion, -2LL = -2 Log Likelihood, df = degrees of freedom. | | | | | |

| *Table SM3f.* Diary WASO and EEG theta power | | | | |  |
| --- | --- | --- | --- | --- | --- |
|  |  | **AIC** | **BIC** | **-2LL** | **df** |
| Theta power | Baseline | 58.21 | 81.20 | 40.21 | 9 |
|  |  | *Age group:* *F*(5,57) = 7.61, *p* <.001** | | |  |
|  | M1 | 47.56 | 71.87 | 27.56 | 10 |
|  |  | *Age group: F*(5,44) = 10.30, *p* <.001** | | |  |
|  |  | *WASO:* *F*(1,39) = 0.99, *p* = .33 | | |  |
|  | M2 | 55.16 | 91.62 | 25.16 | 15 |
|  | M3 | 49.44 | 76.18 | 27.44 | 11 |
| Theta power change (video 1) | Baseline | -57.48 | -35.60 | -75.48 | 9 |
|  |  | *Age group:* *F*(5,73) = 0.63, *p* < .68 | | |  |
|  | M1 | -42.10 | -19.06 | -62.10 | 10 |
|  |  | *Age group: F*(5,51) = 0.36, *p* = .88 | | |  |
|  |  | *WASO:* *F*(1,47) = 1.18, *p* = .28 | | |  |
|  | M2 | -34.59 | -0.03 | -64.59 | 15 |
|  | M3 | -40.10 | -14.75 | -62.10 | 11 |
| *Notes.* *p <.05, **p <.001, °did not survive multiple comparison correction with the Bonferroni method, AIC = Akaike’s Information Criterion, BIC = Schwarz’s Bayesian Criterion, -2LL = -2 Log Likelihood, df = degrees of freedom. | | | | | |

| *Table SM3g.* Diary Night sleep duration and EEG theta power | | | | |  |
| --- | --- | --- | --- | --- | --- |
|  |  | **AIC** | **BIC** | **-2LL** | **df** |
| Theta power | Baseline | 58.21 | 81.20 | 40.21 | 9 |
|  |  | *Age group:* *F*(5,57) = 7.61, *p* <.001** | | |  |
|  | M1 | 47.81 | 72.35 | 27.81 | 10 |
|  |  | *Age group: F*(5,55) = 9.07, *p* <.001** | | |  |
|  |  | *Nigh Sleep Duration:* *F*(1,71) = 0.00, *p* = .96 | | |  |
|  | M2 | 54.21 | 91.22 | 24.21 | 15 |
|  | M3 | 49.56 | 76.56 | 27.56 | 11 |
| Theta power change (video 1) | Baseline | -57.48 | -35.60 | -75.48 | 9 |
|  |  | *Age group:* *F*(5,73) = 0.63, *p* < .68 | | |  |
|  | M1 | -44.70 | -21.39 | -64.70 | 10 |
|  |  | *Age group: F*(5,60) = 0.48, *p* = .79 | | |  |
|  |  | *Night sleep duration:* *F*(1,58) = 0.19, *p* = .66 | | |  |
|  | M2 | -37.12 | -2.16 | -67.12 | 15 |
|  | M3 | -42.78 | -17.14 | -64.78 | 11 |
| *Notes.* *p <.05, **p <.001, °did not survive multiple comparison correction with the Bonferroni method, AIC = Akaike’s Information Criterion, BIC = Schwarz’s Bayesian Criterion, -2LL = -2 Log Likelihood, df = degrees of freedom. | | | | | |

| *Table SM3h.* Diary Day Sleep Duration and EEG theta power | | | | |  |
| --- | --- | --- | --- | --- | --- |
|  |  | **AIC** | **BIC** | **-2LL** | **df** |
| Theta power | Baseline | 58.21 | 81.20 | 40.21 | 9 |
|  |  | *Age group:* *F*(5,57) = 7.61, *p* <.001** | | |  |
|  | M1 | 47.91 | 72.45 | 27.91 | 10 |
|  |  | *Age group: F*(5,52) = 7.05, *p* <.001** | | |  |
|  |  | *Day Sleep Duration:* *F*(1,84) = .25, *p* = .62 | | |  |
|  | M2 | 55.11 | 91.93 | 25.11 | 15 |
|  | M3 | 49.53 | 76.53 | 27.53 | 11 |
| Theta power change (video 1) | Baseline | -57.48 | -35.60 | -75.48 | 9 |
|  |  | *Age group:* *F*(5,73) = 0.63, *p* < .68 | | |  |
|  | M1 | -44.71 | -21.41 | -64.71 | 10 |
|  |  | *Age group: F*(5,58) = 0.52, *p* = .76 | | |  |
|  |  | *Day Sleep Duration:* *F*(1,67) = 0.31, *p* = .58 | | |  |
|  | M2 | -36.52 | -1.56 | -66.52 | 15 |
|  | M3 | -42.73 | -17.09 | -64.73 | 11 |
| *Notes.* *p <.05, **p <.001, °did not survive multiple comparison correction with the Bonferroni method, AIC = Akaike’s Information Criterion, BIC = Schwarz’s Bayesian Criterion, -2LL = -2 Log Likelihood, df = degrees of freedom. | | | | | |

| *Table SM3i.* BISQ Night waking number and EEG theta power | | | | |  |
| --- | --- | --- | --- | --- | --- |
|  |  | **AIC** | **BIC** | **-2LL** | **df** |
| Theta power | Baseline | 58.21 | 81.20 | 40.21 | 9 |
|  |  | *Age group:* *F*(5,57) = 7.61, *p* <.001** | | |  |
|  | M1 | 51.55 | 76.99 | 31.55 | 10 |
|  | M2 | 57.46 | 95.61 | 27.46 | 15 |
|  | M3 | 51.00 | 78.96 | 28.98 | 11 |
|  |  | *Age group: F*(5,54) = 10.26, *p* <.001** | | | |
|  |  | *Night waking number :*  *F*(1,59) = 10.32, *p* = .002* | | | |
|  |  | *Gender F*(1,61) = 2.62, *p* = .11 | | | |
| Theta power change (video 1) | Baseline | -57.48 | -35.60 | -75.48 | 9 |
|  |  | *Age group:* *F*(5,73) = 0.63, *p* < .68 | | |  |
|  | M1 | -55.62 | -31.43 | -75.62 | 10 |
|  |  | *Age group: F*(5,66) = 0.75, *p* = .59 | | |  |
|  |  | *Night waking number :* *F*(1,80) = 1.85, *p* = .18 | | |  |
|  | M2 | -46.63 | -10.35 | -76.63 | 15 |
|  | M3 | -53.81 | -27.20 | -75.81 | 11 |
| *Notes.* *p <.05, **p <.001, °did not survive multiple comparison correction with the Bonferroni method, AIC = Akaike’s Information Criterion, BIC = Schwarz’s Bayesian Criterion, -2LL = -2 Log Likelihood, df = degrees of freedom. | | | | | |

| *Table SM3j.* BISQ WASO and EEG theta power | | | | |  |
| --- | --- | --- | --- | --- | --- |
|  |  | **AIC** | **BIC** | **-2LL** | **df** |
| Theta power 8 | Baseline | 58.21 | 81.20 | 40.21 | 9 |
|  |  | *Age group:* *F*(5,57) = 7.61, *p* <.001** | | |  |
|  | M1 | 58.75 | 84.08 | 38.75 | 10 |
|  |  | *Age group: F*(5,54) = 8.24, *p* <.001** | | |  |
|  |  | *WASO:*  *F*(1,90) = 0.89 *p* = .35 | | |  |
|  | M2 | 66.47 | 104.47 | 36.48 | 15 |
|  | M3 | 59.53 | 87.39 | 37.53 | 11 |
| Theta power change (video 1) | Baseline | -57.48 | -35.60 | -75.48 | 9 |
|  |  | *Age group:* *F*(5,73) = 0.63, *p* < .68 | | |  |
|  | M1 | -56.37 | -32.30 | -76.37 | 10 |
|  |  | *Age group: F*(5,69) = 1.09, *p* = .38 | | |  |
|  |  | *WASO:* *F*(1,79) = 4.40, *p* = .04 | | |  |
|  | M2 | -50.77 | -14.67 | -80.77 | 15 |
|  | M3 | -44.37 | -27.89 | -76.37 | 11 |
| *Notes.* *p <.05, **p <.001, °did not survive multiple comparison correction with the Bonferroni method, AIC = Akaike’s Information Criterion, BIC = Schwarz’s Bayesian Criterion, -2LL = -2 Log Likelihood, df = degrees of freedom. | | | | | |

| *Table SM3k.* BISQ Night sleep duration and EEG theta power | | | | |  |
| --- | --- | --- | --- | --- | --- |
|  |  | **AIC** | **BIC** | **-2LL** | **df** |
| Theta power | Baseline | 58.21 | 81.20 | 40.21 | 9 |
|  |  | *Age group:* *F*(5,57) = 7.61, *p* <.001** | | |  |
|  | M1 | 59.12 | 84.55 | 39.12 | 10 |
|  |  | *Age group: F*(5,54) = 8.11, *p* <.001** | | |  |
|  |  | *Night sleep duration:*  *F*(1,89) = 0.16, *p* = .69 | | |  |
|  | M2 | 60.13 | 98.28 | 30.13 | 15 |
|  | M3 | 59.73 | 87.71 | 37.73 | 11 |
| Theta power change (video 1) | Baseline | -57.48 | -35.60 | -75.48 | 9 |
|  |  | *Age group:* *F*(5,73) = 0.63, *p* < .68 | | |  |
|  | M1 | -54.38 | -30.19 | -74.38 | 10 |
|  |  | *Age group: F*(5,74) = 0.58, *p* = .71 | | |  |
|  |  | *Night sleep duration:* *F*(1,70) = 0.120, *p* = .82 | | |  |
|  | M2 | -48.29 | -12.01 | -78.29 | 15 |
|  | M3 | -52.46 | -25.85 | -74.46 | 11 |
| *Notes.* *p <.05, **p <.001, °did not survive multiple comparison correction with the Bonferroni method, AIC = Akaike’s Information Criterion, BIC = Schwarz’s Bayesian Criterion, -2LL = -2 Log Likelihood, df = degrees of freedom. | | | | | |

| *Table SM3l.* BISQ Day Sleep Duration and EEG theta power | | | | |  |
| --- | --- | --- | --- | --- | --- |
|  |  | **AIC** | **BIC** | **-2LL** | **df** |
| Theta power | Baseline | 58.21 | 81.20 | 40.21 | 9 |
|  |  | *Age group:* *F*(5,57) = 7.61, *p* <.001** | | |  |
|  | M1 | 59.26 | 84.69 | 39.26 | 10 |
|  |  | *Age group: F*(5,53) = 7.60, *p* <.001** | | |  |
|  |  | *Sleep quality:*  *F*(1,88) = 0.02, *p* = .90 | | |  |
|  | M2 | 63.78 | 101.93 | 33.78 | 15 |
|  | M3 | 59.84 | 87.82 | 37.84 | 11 |
| Theta power change (video 1) | Baseline | -57.48 | -35.60 | -75.48 | 9 |
|  |  | *Age group:* *F*(5,73) = 0.63, *p* < .68 | | |  |
|  | M1 | -54.14 | -30.00 | -74.14 | 10 |
|  |  | *Age group: F*(5,71) = 0.68, *p* = .64 | | |  |
|  |  | *Day Sleep Duration:* *F*(1,75) = 0.161, *p* = .69 | | |  |
|  | M2 | -41.81 | 6.32 | -81.81 | 15 |
|  | M3 | -52.22 | -25.62 | -74.22 | 11 |
| *Notes.* *p <.05, **p <.001, °did not survive multiple comparison correction with the Bonferroni method, AIC = Akaike’s Information Criterion, BIC = Schwarz’s Bayesian Criterion, -2LL = -2 Log Likelihood, df = degrees of freedom. | | | | | |

**Section 6** – **Association of theta power and Ages & Stages Questionnaire**

Below an illustration of the association between theta parameters and the developmental questionnaire Ages & Stages.

| *Table SM4.* Association of theta power and Ages & Stages Questionnaire | | | |
| --- | --- | --- | --- |
|  |  | Overall theta power | Theta change |
| ASQ Communication Subscale | r | .043 | .102 |
|  | P-value | .753 | .457 |
| ASQ Gross Motor Subscale | r | .017 | .077 |
|  | P-value | .902 | .572 |
| ASQ Fine Motor Subscale | r | .160 | -.066 |
|  | P-value | .239 | .629 |
| ASQ Problem Solving Subscale | r | .077 | .092 |
|  | P-value | .572 | .502 |
| ASQ Social Subscale | r | .284 | -.239 |
|  | P-value | .034 | .076 |

*Note.* Partial correlations controlled by age in days; significant p-value disappear after controlling for multiple comparison.

**Section 7 – Sleep diary
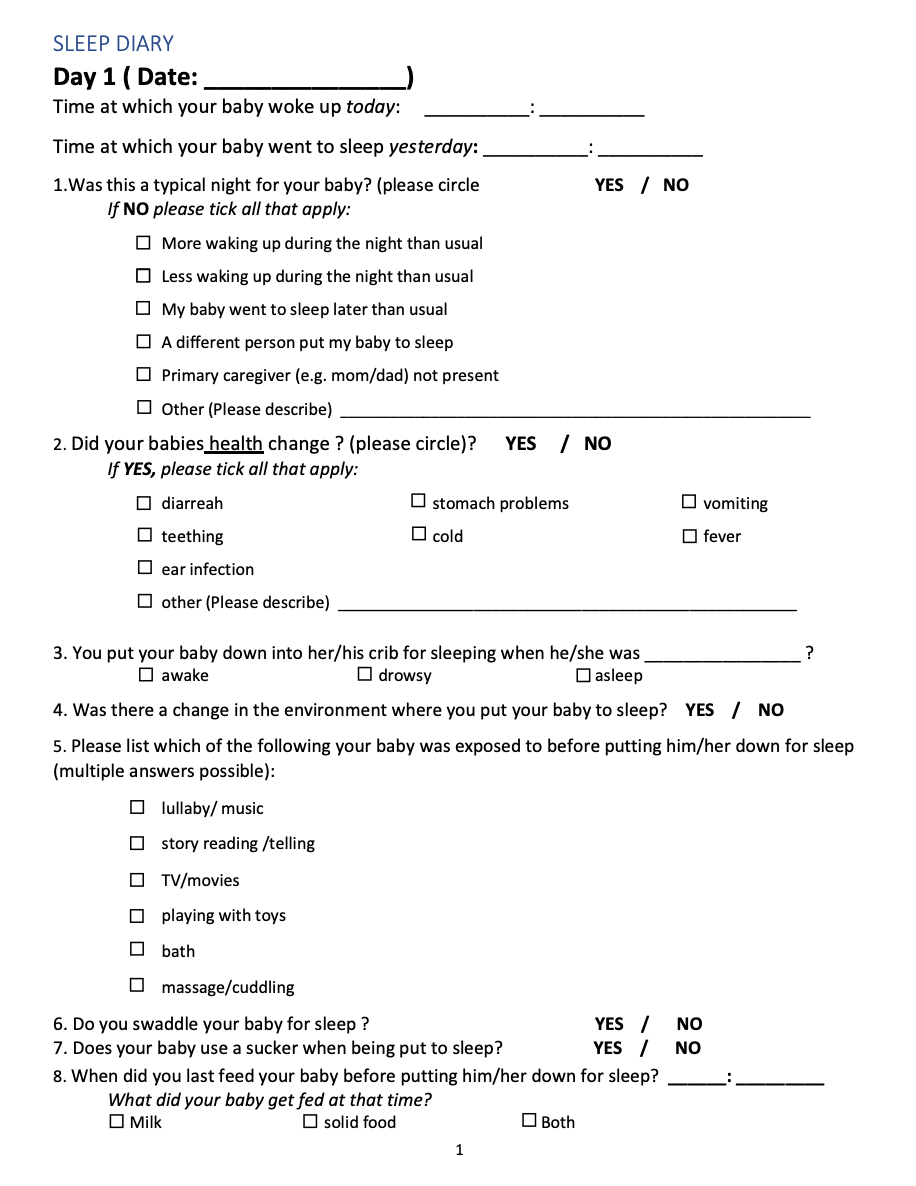

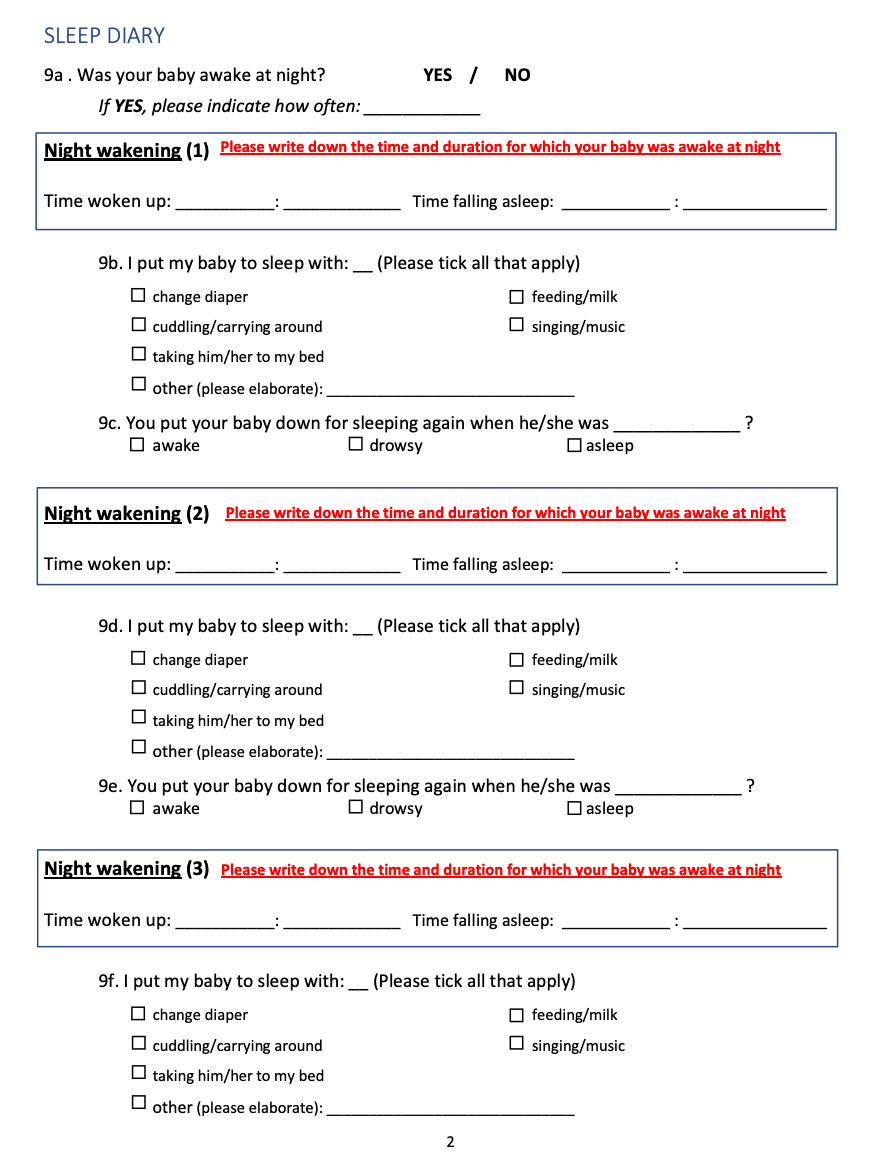
**

**
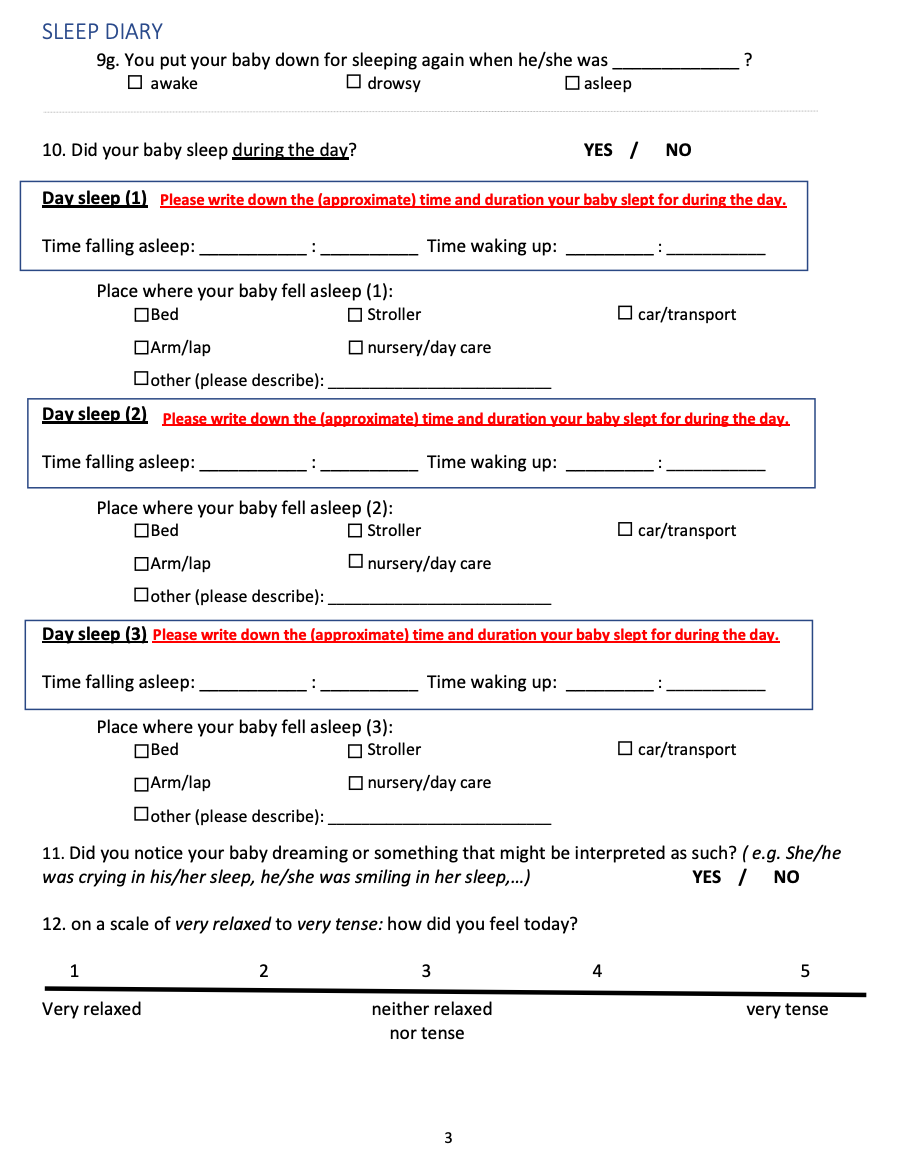
**
